# Treatment of Hepatocellular Carcinoma by Intratumoral Injection of ^125^I-AA98 mAb and Its Efficacy Assessments by Molecular Imaging

**DOI:** 10.3389/fbioe.2019.00319

**Published:** 2019-11-14

**Authors:** Jun Zhou, Pengcheng Hu, Zhan Si, Hui Tan, Lin Qiu, He Zhang, Zhequan Fu, Wujian Mao, Dengfeng Cheng, Hongcheng Shi

**Affiliations:** ^1^Department of Nuclear Medicine, Zhongshan Hospital, Fudan University, Shanghai, China; ^2^Department of Nuclear Medicine, Xuhui District Central Hospital of Shanghai, Shanghai, China; ^3^Shanghai Institute of Medical Imaging, Shanghai, China

**Keywords:** AA98 mAb, hepatocellular carcinoma, CD146, ^125^I, angiogenesis, apoptosis

## Abstract

**Objective:** To investigate the therapeutic efficacy of intratumoral injection of ^125^I-AA98 mAb for hepatocellular carcinoma (HCC) and its therapy efficacy assessment by ^99m^Tc-HYNIC-duramycin and ^99m^Tc-HYNIC-3PRGD2 SPECT/CT imaging.

**Methods:** HCC xenograft tumor mice models were injected intratumorally with a single dose of normal saline, 10 microcurie (μCi) ^125^I-AA98 mAb, free ^125^I, AA98 mAb, 80 μCi ^125^I-AA98 mAb, and 200 μCi ^125^I-AA98 mAb. ^99m^Tc-HYNIC-duramycin and ^99m^Tc-HYNIC-3PRGD2 micro-SPECT/CT imaging were performed on days 3 and 7, respectively. The T/M ratio for each imaging was compared with the corresponding immunohistochemical staining at each time point. The relative tumor inhibition rates were documented.

**Results:** In terms of apoptosis, the 200 μCi group demonstrated the highest apoptotic index (11.8 ± 3.8%), and its T/M ratio achieved by ^99m^Tc-HYNIC-duramycin imaging on day 3 was higher than that of the normal saline group, 80 μCi group, 10 μCi group and free ^125^I group on day 3, respectively (all *P* < 0.05). On day 3, there was a markedly positive correlation between T/M ratio from ^99m^Tc-HYNIC-duramycin imaging and apoptotic index by TUNEL staining (*r* = 0.6981; *P* < 0.05). Moreover, the 200 μCi group showed the lowest T/M ratio on ^99m^Tc-HYNIC-3PRGD2 imaging (1.0 ± 0.5) on day 7 (all *P* < 0.05) comparing to other groups. The T/M ratio on day 7 was not correlated with integrin α_ν_β_3_ staining (*P* > 0.05). The relative inhibitory rates of tumor on day 14 in the AA98 mAb, 10 μCi, 80 μCi, free ^125^I, and 200 μCi groups were 26.3, 55.3, 60.5, 66.3, and 69.5%, respectively.

**Conclusion:** ^125^I-AA98 mAb showed more effective apoptosis induced ability for CD146 high expression Hep G2 HCC cells and hold the potential for HCC treatment. Moreover, ^99m^Tc-HYNIC-Duramycin (apoptosis-targeted) imaging and ^99m^Tc-HYNIC-3PRGD2 (angiogenesis-targeted) imaging are reliable non-invasive methods to evaluate the efficacy of targeted treatment of HCC.

## Introduction

Hepatocellular carcinoma (HCC) is the most common primary malignant tumor of the liver in adults (Torre et al., 2015). The majority of patients diagnosed with advanced HCC and failed to meet the criteria for resection or transplantation (Thomas et al., 2010). Although locoregional therapies including transarterial chemoembolization, cryotherapy, and radiofrequency or microwave ablation can reduce tumor burden while preserving liver function, the prognosis of advanced HCC patients still remains dismal. Alternatively, intratumoral injection of radiopharmaceuticals can achieve a high therapeutic concentration at targeted sites with an extended period of time and avoid systemic exposure to radiation and the results are promising (Tian et al., 1996; Wang et al., 1998; Junfeng et al., 2000; Chi et al., 2014).

It is widely accepted that angiogenesis is essential for tumor growth and invasion (Folkman, 1971). HCC is one of the malignant hypervascular solid tumors, and inhibition of angiogenesis is one of the important methods to treat HCC. CD146 has emerged as a biomarker for angiogenesis (Zheng et al., 2009; Wang and Yan, 2013; Nomikou et al., 2015), and it has been identified as an attractive target for imaging and therapy in HCC (Thomann et al., 2014; Hernandez et al., 2016). Furthermore, high expression of CD146 predicted poor prognosis in HCC patients (Jiang et al., 2016). AA98 monoclonal antibody (mAb) is a promising mAb against CD146 by inhibition of angiogenesis and tumor growth, but it does not induce apoptosis *in vitro* (Yan et al., 2003). Anti-angiogenic mono-therapy is a powerful tool to inhibit tumor growth, but, to date, any available anti-angiogenic mono-therapies targeting cancer do not satisfy the expectation of “starving the tumor.”

One of the solutions to enhance this anti-angiogenic efficacy is to label the anti-angiogenic agents with therapeutic radionuclides, and this strategy is supported by previous reports (Tijink et al., 2006; Fujiwara et al., 2014; Park et al., 2017; Ehlerding et al., 2018). Iodine-125 (^125^I) is a long-lived radioisotope with a half-life of 59.5 days and a short-range emitter (Cunningham et al., 1998). Its decay can produce a highly localized deposition of dose by short-range Auger electrons plus X ray and Gamma ray. ^125^I can cause non-repairable damage to double strand DNA and then induce tumor cell death with favorable results by several possible mechanisms including apoptosis hypothesis (Hofer and Hughes, 1971; Bagshawe et al., 1991; Cunningham et al., 1998).

Molecular imaging plays a critical role in monitoring tumor response to targeted therapy. ^99m^Tc is an easily accessible diagnostic radionuclide using ^99^*Mo*/^99m^Tc generator system, and it has a very attractive nuclear property for molecular imaging. The main targets for apoptosis are the two common cell membrane aminophospholipids, such as the richest phosphatidylserine (PS) and the second richest phosphatidylethanolamine (PE). PE can be recognized and bound with high affinity by duramycin with a 19-amino acid peptide during cell apoptosis and necrosis (Zhao, 2011). Moreover, the stable, fast clearing, and highly specific ^99m^Tc-HYNIC-duramycin can be applied to detect tumor cell death and monitor tumor response to treatment (Elvas et al., 2015; Hu et al., 2018). Integrin α_ν_β_3_ is an attractive molecular target for its highly restricted expression in tumor angiogenesis and metastasis. The ^99m^Tc-HYNIC-3PRGD2 (3PEG4-cRGD dimer) with a safe, highly stable, rapid blood clearance, and easily available kit formulation has the potential for non-invasive integrin α_ν_β_3_-targeted imaging and monitoring treatment efficacy of antiangiogenesis (Wang et al., 2009; Jia et al., 2011).

In the present study, AA98 mAb was labeled with ^125^I to treat HCC xenograft by intratumoral injection. In the following, apoptosis-targeted imaging with ^99m^Tc-HYNIC-duramycin and angiogenesis-targeted imaging with ^99m^Tc-HYNIC-3PRGD2 were employed to evaluate treatment efficacy.

## Materials and Methods

### Preparation of ^125^I-AA98 mAb and Quality Control

The modified iodination protocol of AA98 mAb with ^125^I was employed fundamentally as described previously (Visser et al., 2001; Tijink et al., 2009). Briefly, 800 μg AA98 mAb (80 μL, 10 mg/mL) was added into a tube coated with 25 μg Iodogen (Pierce Biotechnology), then 0.5-μL Na^125^I solution (GMS Pharmaceutical Co., Ltd., Shanghai, China) with specific activity of 1.6 Ci/mL was added. After 10 min, the reaction was terminated. The radiochemical purity (RCP) of ^125^I-AA98 mAb was assessed by radio-thin layer chromatography (radio-TLC) Imaging Scanner (Eckert & Ziegler Radiopharma, Inc., USA).

### Preparation of ^99m^ Tc-Labeled Probes for Tumor Apoptosis and Angiogenesis

A single-step kit containing 15 μg hydrazinonicotinamide (HYNIC)-duramycin (Molecular Targeting Technologies, Inc., USA) was labeled by Na^99m^TcO_4_ as previously reported (Zhao et al., 2008; Hu et al., 2018). In brief, 0.5 mL freshly prepared sodium pertechnetate (^99m^Tc-pertechnetate) with specific activity of 30 millicurie (mCi), which was obtained from Mo-99/Tc-99m generator (GMS Pharmaceutical Co., Ltd., Shanghai, China), was added to the kit vial. The vial was heated at 80°C around 20 min and then cooled down to room temperature. The RCP of the ^99m^Tc-HYNIC-duramycin (always beyond 95%) was analyzed by Radio-TLC.

A single-step kit containing 20 μg of the HYNIC-3PRGD2 conjugate was labeled by Na^99m^TcO_4_ as previously reported (Wang et al., 2009). Simply put, 1.0 mL of Na^99m^TcO_4_ solution (20 mCi) in saline was added into the kit vial and heated at 100°C around 20 min. After heating, the vial was cooled down to room temperature. The RCP of the ^99m^Tc-HYNIC-3PRGD2 was >95%.

### Cell Lines

The human HCC cell line Hep G2 was obtained from the Cell Bank, Shanghai Institutes for Biological Sciences, Chinese Academy of Sciences, Shanghai. Hep G2 cells were cultured in DMEM medium (Gibco) containing 1% penicillin-streptomycin (100 U/mL penicillin; 100 μg/mL streptomycin) and 10% fetal bovine serum at 37°C with 5% CO_2_. All cells were passaged and collected with trypsin-EDTA solution (0.05% trypsin; 0.02% EDTA).

### Animal Care

All animal studies were performed under a protocol approved by the Institutional Animal Care and Use Committee of Zhongshan Hospital, Fudan University. All mice were acclimatized to laboratory conditions (individually ventilated cages, 20°C, 12/12 h light/dark cycle, and *ad libitum* to a standard diet) around 1 week prior to studies.

### Animal Model and Intratumoral Injection

Female athymic BALB/c nu/nu mice (6–8 weeks, Shanghai SLAC Laboratory Animal Co., Ltd.) were injected s.c. into the right shoulder with Hep G2 cells (3 × 10^5^) in 0.1 mL phosphate buffer solution (PBS). When tumors reached 6–10 mm in diameter (around 3 weeks after inoculation), 0.25% sodium iodide was feed to mice for 3 days to block the absorption of ^125^I by the thyroid gland before intratumoral injection, and this treatment lasted until the end of experiments. Sixty-six athymic mice bearing s.c. Hep G2 tumor xenografts were randomized into six groups (*n* = 11/group) to monitor tumor apoptosis (*n* = 3/group), evaluate angiogenesis (*n* = 3/group), and observe therapeutic efficacy (*n* = 5/group). All mice received a single intratumoral injection in a volume up to 20 μL as follows: control (normal saline, 20 μL/mouse), 10 microcurie (μCi) ^125^I-AA98 mAb (10 μCi ^125^I-10 μg AA98 mAb/mouse), free ^125^I (80 μCi ^125^I/mouse), AA98 mAb (80 μg/mouse), 80 μCi ^125^I-AA98 mAb (80 μCi ^125^I-80 μg AA98 mAb/mouse), and 200 μCi ^125^I-AA98 mAb (200 μCi ^125^I-200 μg AA98 mAb/mouse). The injection was administered slowly into the center of the tumor using a microsyringe. The needle was left in the tumor for several seconds before withdrawal, and the injection site was pressed using cotton ball around 1 min.

### *In vivo* Micro-SPECT/CT Imaging

Prior to micro-SPECT/CT imaging, mice were placed into a mice anesthesia chamber with 4% isoflurane mixed with 2 L/min O_2_ for several minutes to induce deep anesthesia using a VETEQUIP V-1 animal anesthesia system (VetEquip Inc., USA), then mice were transferred to exam table and maintained with 1.5% isoflurane mixed with 0.4 L/min O_2_ using the same anesthesia system. The Nano SPECT/CT scanner (Bioscan, USA) was employed to perform *in vivo* micro-SPECT/CT imaging with the settings (Tan et al., 2017). All 3D OSEM images were reconstructed with a HiSPECT algorithm.

Tumor responses in the tumors were evaluated with ^99m^Tc-HYNIC-duramycin and ^99m^Tc-HYNIC-3PRGD2. Before treatment (day 0), baseline micro-SPECT/CT imaging was performed in each of imaging groups using the two molecular probes. After intratumoral injections, micro-SPECT/CT imaging targeting tumor apoptosis was performed using ^99m^Tc-HYNIC-duramycin on day 3, and targeting tumor angiogenesis using ^99m^Tc-HYNIC-3PRGD2 on day 7.

### Data Analysis

All micro-SPECT/CT images were displayed with Invivo Scope software (Version 1.43, Bioscan, USA). An appropriate 3D volume of interest (VOI) was placed in the tumor with maximum molecular probe uptake included and necrotic area spared, and the contralateral muscle area was also measured. The radioactivity of tumor or contralateral muscle (in μCi/mm^3^) was reported by the software for each VOI. The tumor-to-contralateral muscle tissues ratio (T/M ratio) was calculated for each tumor. For assessment of tumor apoptosis and angiogenesis, the T/M ratio of each of the six groups after intratumoral injection was compared with that of the corresponding baseline imaging, and multiple comparisons of the T/M ratio were also conducted among the six groups after intratumoral injection.

### Therapeutic Efficacy Study

Tumor size (length and width in mm) and body weight (gram) were measured every other day for 14 days after injection. Tumor volume (mm^3^) was calculated following the formula (Inaba et al., 1989): Volume = [length × (width)^2^]/2. The relative tumor inhibition rate was used to assess the antitumor efficacy of the different treatments as follows:

$$\begin{array}{l} \text{The relative tumor inhibition rate}=[\text{1}-{\left({\text{averageV}}_{\text{14}}{\text{/V}}_{\text{0}}\right)}_{\text{T}}\text{/}\\ \;{\left({\text{averageV}}_{\text{14}}{\text{/V}}_{\text{0}}\right)}_{\text{C}}]\times 100\%. \end{array}$$

Where V_14_ is the tumor volume on Day 14, V_0_ is the pretreatment tumor volume before injection (Day 0), T represents each of the five treatment groups, and C means the control group. When body weight loss was >20%, or tumor volume was larger than 2 cm^3^, mice were sacrificed and excluded to calculate the relative tumor inhibition rate.

### Histiopathological and Immunohistochemistry

After imaging or treatment, all mice were sacrificed, and all tumors were harvested. Adjacent 4 μm thick paraffin-embedded sections of all tumor samples were stained with Haematoxylin and Eosin (HE) and terminal deoxynucleotidyl transferase (TdT)-mediated dUTP nick end labeling (TUNEL) fluorescence or integrin α_ν_β_3_ fluorescence. In brief, all tumors were fixed in 4% paraformaldehyde in PBS, dehydrated, and embedded in paraffin, then sliced in the maximal section of the tumor. According to the instruction of the *in situ* cell death detection kit (Roche), TUNEL fluorescence staining was performed to detect and quantify tumor cell apoptosis. To stain integrin α_ν_β_3_, the 4 μm slices were blocked with 10% goat serum at 37°C for 20 min. The sections were then incubated with rabbit anti-integrin α_ν_β_3_ antibody (1:200, Beijing Biosynthesis Biotechnology Co., Ltd.) overnight at 4°C. Then, the sections were visualized with Cy3-labeled goat anti-rabbit antibody (1:200, Abcam). Finally, tumor sections were mounted in 4′, 6-diamidino-2-phenylindole dihydrochloride (DAPI) mounting medium.

### Pathological and Immunohistochemistry Analysis

The percentage of tumor necrotic area was calculated as: the area of tumor necrosis/the area of total tumor tissue (drawn manually) on HE sections using Case Viewer (3dhistech Ltd., Hungary). Apoptosis index (AI) was determined by calculating the percentage of apoptotic nuclei with green TUNEL fluorescence staining over total nuclei using Quant Center software (3dhistech Ltd., Hungary). Image-Pro Plus 6.0 software (Media Cybernetics, USA) was used to assess the integrin α_ν_β_3_ red fluorescence intensity by measuring the integrated optical density per field of view (IOD/mm^2^) of the images with an equivalent area.

### Statistics

The numerous data were expressed as mean ± standard deviation. The results were analyzed usinig the software package (IBM SPSS version 22.0 for Mac OS, IBM, USA). A *P* < 0.05 was considered to indicate statistically significant difference.

## Results

The labeling rate of the ^125^I-AA98 mAb molecular probe was over 95%. Both the radiochemical purity and the radiochemical yield were 95.8 ± 0.5% (Figure S1).

### Apoptosis Imaging

**Figure 1:**
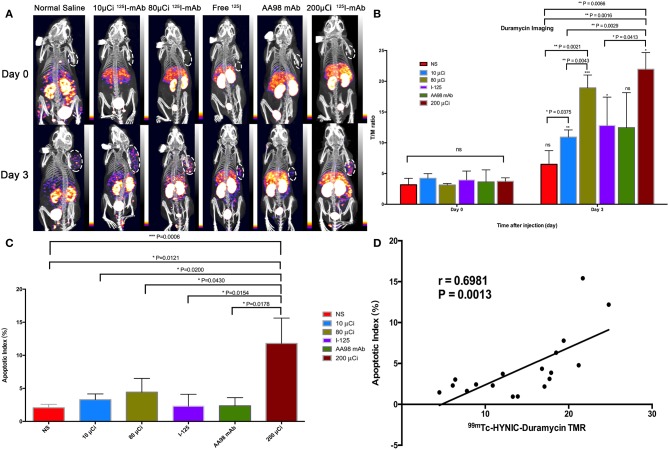
*In vivo* SPECT/CT imaging with ^99m^Tc-HYNIC-Duramycin and apoptosis immunofluorescence staining in HCC xenograft mice models. Tumor images were obtained with SPECT/CT before (upper row) and 3 days after (bottom row) intratumoral injection among the six groups **(A)**. **(B)** T/M ratio was analyzed from ^99m^Tc-HYNIC-Duramycin imaging at baseline scan before intratumoral injection and second scan 3 days after intratumoral injection. **(C)** Correlation between T/M ratio and apoptosis index. **(D)** Multiple comparisons of average apoptosis index were performed among the six groups. [Note-^125^I-mAb means ^125^I-AA98 mAb; *n*=3, mean ± SD, *P < 0.05; **P < 0.01; ***P < 0.001; ns indicates no significance between the baseline scan on day 0 and the second scan on day 3, *P* > 0.05; μCi means microcurie; white dashed circled area in **(A)** indicates HCC tumor].

**Figure 2:**
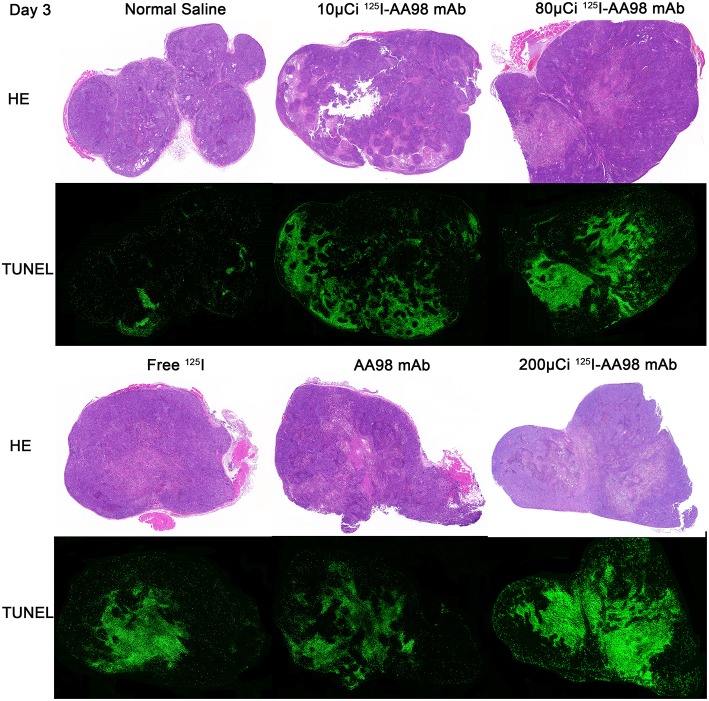
Photographs of pair-matched HE staining and TUNEL fluorescence staining 3 days after intratumoral injection in each group.

In terms of T/M ratio, ^99m^Tc-HYNIC-Duramycin imaging showed that the baseline scans had no statistically significant difference (*P* > 0.05, Figure 1). Three days after treatment, the T/M ratio in each group was higher than that before treatment. The T/M ratio of the 10 μCi group, 80 μCi group, free ^125^I group, and 200 μCi group on day 3 was higher than that on the corresponding baseline scans (*P* = 0.0011, *P* = 0.0002, *P* = 0.0332, and *P* = 0.0218, respectively). There was statistically significant difference among all the 6 groups on day 3 (*P* = 0.0066). In terms of the degree of apoptosis, the 200 μCi group demonstrated the highest apoptotic index (11.8 ± 3.8%), and its T/M ratio on day 3 was higher than that of the normal saline, 10 μCi and free ^125^I groups, respectively (*P* = 0.0016, *P* = 0.0029, and *P* = 0.0413, respectively). The T/M ratio in 80 μCi group on day 3 was higher than that in 10 μCi and normal groups (*P* = 0.0021 and *P* = 0.0043). In terms of T/M ratio on day 3, there was also a markedly significant difference between 10 μCi group and normal group (*P* = 0.0375). There were no significant differences among the remaining comparative studies (all *P* > 0.05). There was a markedly positive correlation (*r* = 0.6981; *P* = 0.0013) between T/M ratio and apoptotic index by TUNEL fluorescence staining (Figure 2) on day 3.

### Angiogenesis Imaging

**Figure 3:**
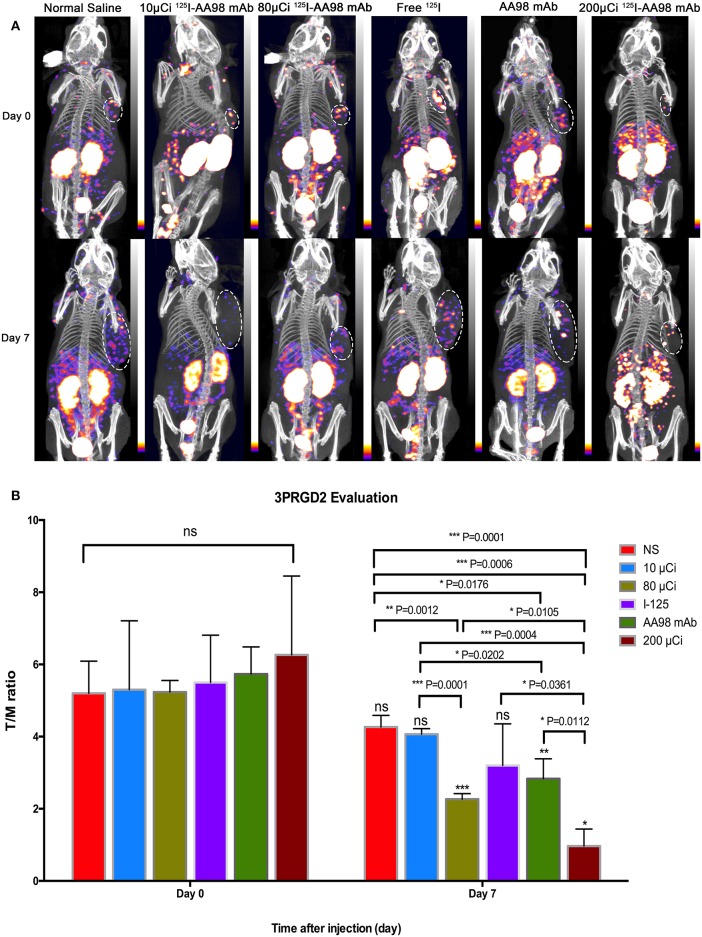
*In vivo* SPECT/CT imaging with ^99m^Tc-HYNIC-3PRGD2 and integrin α_ν_β_3_ immunofluorescence staining in HCC xenograft mice models. Tumor images were obtained with SPECT/CT before (upper row) and 7 days after (bottom row) intratumoral injection among the six groups **(A)**. **(B)** T/M ratio was analyzed from ^99m^Tc-HYNIC-3PRGD2 imaging at baseline scan before intratumoral injection and second scan 7 days after intratumoral injection. (*n* = 3, mean ± SD, *P < 0.05; **P < 0.01; ***P < 0.001; ns indicates no significance between the baseline scan on day 0 and the second scan on day 7, *P* > 0.05; μCi means microcurie; white dashed circled area in **(A)** indicates HCC tumor).

**Figure 4:**
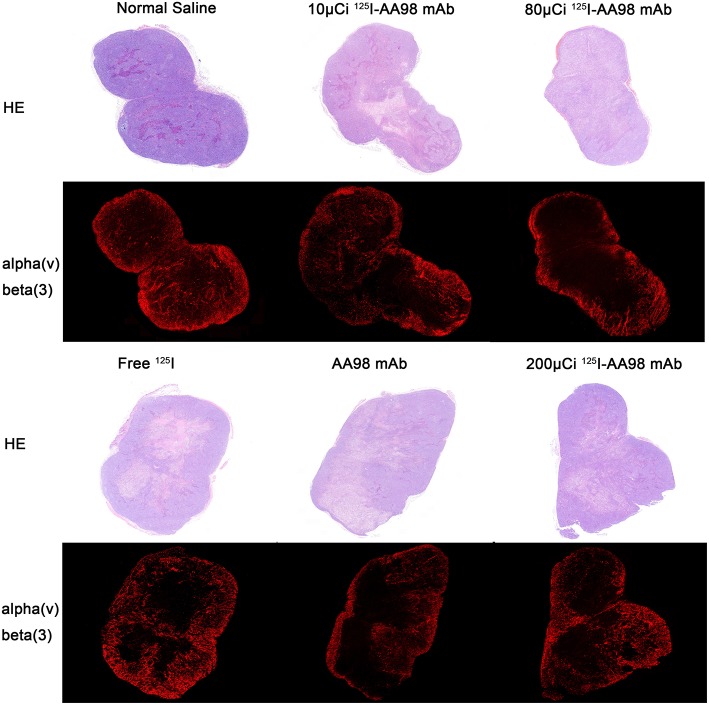
Photographs of pair-matched HE staining and integrin α_ν_β_3_ fluorescence staining 7 days after intratumoral injection in each group.

In terms of T/M ratio, ^99m^Tc-HYNIC-3PRGD2 imaging showed there was no statistically significant difference among the baseline scans (*P* > 0.05) (Figure 3). Seven days after treatment, the T/M ratio was significantly lower than that before treatment, and there had no statistically significant difference among all groups (*P* = 0.0001). There was marked difference between the baseline scan and the repeated scan on day 7 in 200 μCi group, 80 μCi group, and AA98 mAb group, respectively (all *P* < 0.05). The 200 μCi group showed the lowest T/M ratio (1.0 ± 0.5) compared with the other five groups on day 7 (all *P* < 0.05). The T/M ratio in 80 μCi group and AA98 mAb group on day 7 were both higher than that in 10 μCi group and saline control group on day 7 (all *P* < 0.05). The T/M ratio of angiogenesis-targeted imaging on day 7 was not correlated with IOD/mm^2^ by integrin α_ν_β_3_ immunofluorescence staining (Figure 4) (*P* = 0.1020).

### Therapeutic Efficacy

**Figure 5:**
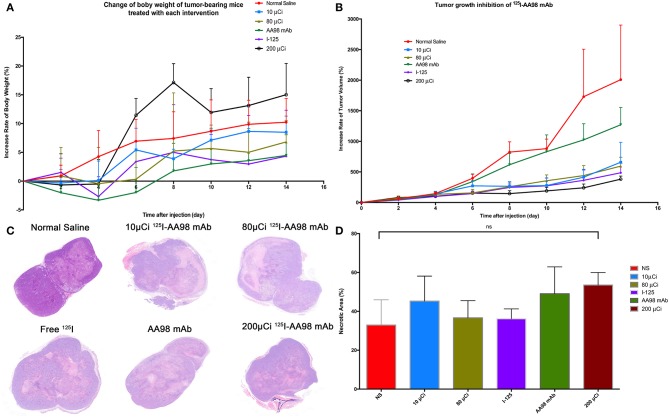
Fourteen days surveillance after intratumoral injection among the six groups and HE staining in HCC xenograft mice models. **(A)** Change of percentage of body weight in HCC xenograft mice models was calculated. **(B)** Change of percentage of tumor size in HCC xenograft mice models was documented. **(C)** Photographs of HE staining for HCC tumor 7 days after intratumoral injection in each group. **(D)** Multiple comparisons of average necrotic area of HCC tumor were performed among the six groups.

As shown in Figure 5, compared with the control group, the relative inhibitory rates of tumor in AA98 mAb group, 10 μCi group, 80 μCi group, ^125^I group, and 200 μCi group on day 14 were 26.3%, 55.3%, 60.5%, 66.3%, and 69.5%, respectively. Fourteen days after intratumoral injection, there is no markedly statistical difference in terms of tumor volume growth rate among all the six groups (*P* > 0.05). No significant difference of the percentage of tumor necrotic area was noted among the six groups (*P* > 0.05).

## Discussion

CD146 is one of adhesion molecules involving in angiogenesis (St Croix et al., 2000; Chan et al., 2005), and it also plays an essential role in tumor progression. Recently, overexpression of CD146 was found to promote migration and invasion of HCC cells and predict poor prognosis in HCC patients (Jiang et al., 2016). A novel anti-CD146 mAb AA98 has been revealed that it can block angiogenesis both *in vitro* and *in vivo*, and it alone can effectively inhibit tumor growth of HCC xenografts via intraperitoneal injections (Yan et al., 2003). In clinical settings, the usual way of treating inoperable HCC is transcatheter arterial chemoembolization (TACE), which is not easily accessible for HCC xenograft mice model. Alternatively, intratumoral injection is preferred to mimic TACE as a way of drug delivery.

AA98 mAb, as an angiogenesis inhibitor, can block the formation of new blood vessel, rather than directly damage existing blood vessel to result in secondary tumor cell apoptosis (Siemann and Horsman, 2009). Previous report also showed AA98 mAb could not directly induce cell apoptosis *in vitro* (Yan et al., 2003). However, ^125^I can delivery longer short-range internal radiation over time to induce tumor cell apoptosis. One of the possible molecular mechanisms of tumor cell apoptosis that ^125^I may upregulate expression of p53 to downregulate vascular endothelial growth factor and then decrease microvessel density (Ma et al., 2014). We were motivated to try the intratumoral injection of ^125^I-AA98 mAb to target CD146 with AA98 mAb and then to induce HCC cell apoptosis by ^125^I with its potential mechanisms (Cunningham et al., 1998; Ma et al., 2014).

In our experiments, AA98 mAb was labeled with a high specific activity of Na^125^I solution by Iodogen method to yield >95% of RCP. Compared with the baseline T/M ratio of *in vivo* ^99m^Tc-HYNIC-Duramycin imaging before intratumoral injection, the T/M ratio of the four groups containing ^125^I revealed marked increase of HCC cell apoptosis after 3-day treatment (*P* < 0.05), but not in AA98 mAb group (*P* > 0.05). Noticeable, ^125^I-AA98 mAb for targeting CD146 might more efficiently induce apoptosis than free ^125^I. The central HCC tumor apoptosis with peripheral normal HCC cells among all groups was verified by TUNEL staining. For *in vivo* ^99m^Tc-HYNIC-Duramycin imaging on day 3, the T/M ratios of the 3 kinds of molecular probe groups (10 μCi group, 80 μCi group, and 200 μCi group) were significantly different from that of the control group (*P* < 0.05), but not the free ^125^I group (*P* > 0.05). Compared with the control group before treatment, although there was slight increase tendency of ^99m^Tc-HYNIC-Duramycin uptake within tumor in the 3 kinds of molecular probe groups on day 3 (*P* > 0.05), the inherently spontaneous HCC tumor apoptosis in the three kinds of molecular probe groups was ruled out.

We also confirmed that ^125^I low-energy radionuclide which resulting a highly localized irradiation can kill HCC cells via apoptosis-mediated cell death. According to dosage of ^125^I, there is a tendency that higher dosage may induce higher apoptosis. Moreover, the 200 μCi group alone had the highest apoptotic index and markedly different from the five other groups (all *P* < 0.05). It means that higher dosage of ^125^I-AA98 mAb will cause more serious apoptosis, and the 200 and 80 μCi groups may superior to the free ^125^I group. Finally, our results revealed that the T/M ratio of *in vivo* ^99m^Tc-HYNIC-Duramycin imaging had a positive correlation with apoptotic index by TUNEL fluorescence staining. It indicates that *in vivo* ^99m^Tc-HYNIC-Duramycin imaging can provide a potential tool to evaluate tumor cell apoptosis.

AA98 mAb plays an essential role in inhibiting early angiogenic processes, which control the formation of secondary and tertiary vessel branches (Yan et al., 2003). Tumor neoangiogenesis was able to be identified by overexpression of the integrin α_ν_β_3_ receptor too, which can be visualized by radionuclide-labeled RGD analogs, such as the molecular probe ^99m^Tc-HYNIC-3PRGD2 (Jin et al., 2016). We found that the groups containing 80 μg of AA98 mAb or more (i.e., 200 μCi group, 80 μCi group, and AA98 mAb group) showed marked difference between the baseline scan on day 0 and the repeated scan on day 7 (all *P* < 0.05). This finding indicates that AA98 mAb of ≥ 80 μg may be the optimal single dosage to achieve response detected on ^99m^Tc-HYNIC-3PRGD2 SPECT/CT imaging during 7-day therapy. These results were consistent with previous report (Yan et al., 2003). However, our study found that only 200 μCi group was superior to the other five groups (*P* < 0.05), although the T/M ratio of *in vivo* ^99m^Tc-HYNIC-3PRGD2 SPECT/CT imaging on day 7 was not correlated with IOD/mm^2^ by integrin α_ν_β_3_ immunofluorescence staining (*P* > 0.05). The fixed dosage of AA98 mAb may be one of the reasons to limit the anti-angiogenetic effects. Further work is needed to apply multiple injections for treatment to achieve better response.

In addition, our data also showed that there is a tendency of inhibiting growth of HCC xenografts using 125I-AA98 mAb for the first time, although there is no marked significant difference among the five treatment groups (*P* > 0.05). We noted that the relative inhibitory rate of tumor in the ^125^I group was very well (66.3%). However, the average necrotic area in the ^125^I group was just higher than the in the control group and lower than that in the other four groups. It indicates that the percentage of the tumor tissue in the ^125^I group was only lower than that in the control group. This is the first point that we thought the ^125^I group did not share the best anti-tumor effect. Second, the average apoptotic index in the ^125^I group on day 3 also showed it is inferior to the three groups containing ^125^I-AA98 mAb. Third, the evaluation of the SPECT/CT imaging in terms of anti-apoptosis and anti-angiogenesis demonstrated that the tumor-muscle rates did not indicate the ^125^I group was the best one. Based on our study, we think our further research may focus on comparing the ^125^I-AA98 mAb with free ^125^I, optimal multiple injections and optimal injection interval to confirm the definite efficacy.

In conclusion, compared with free ^125^I and unlabeled AA98 mAb, it's evident that ^125^I-AA98 mAb showed more effective apoptosis induced ability for CD146 high expression Hep G2 HCC cells. Moreover, ^99m^Tc-HYNIC-Duramycin (apoptosis-targeted) imaging and ^99m^Tc-HYNIC-3PRGD2 (angiogenesis-targeted) imaging are reliable non-invasive methods to evaluate the efficacy of targeted treatment of HCC.

## Data Availability Statement

The datasets generated for this study are available on request to the corresponding author.

## Ethics Statement

The animal study was reviewed and approved by the Animal Care Committee of Fudan University.

## Author Contributions

JZ, PH, and DC were involved in the study data analysis and edited the main manuscript text. LQ, HZ, ZF, and JZ contributed to animal models. ZS and DC helped in synthesizing the probe used in this study. HT and WM deal with the pathology. JZ, PH, DC, and HS conceived and designed as well as controlled the quality of this study.

### Conflict of Interest

The authors declare that the research was conducted in the absence of any commercial or financial relationships that could be construed as a potential conflict of interest.

## Supplementary Material

Figure S1 The radiochemical purity (96.3%) of ^125^I-AA98 mAb was assessed by radio-thin layer chromatography.

## Abbreviations

AI: apoptosis index
DAPI: 4′ , 6-diamidino-2-phenylindole dihydrochloride
HCC: hepatocellular carcinoma
HE: haematoxylin and eosin
HYNIC: mAb, monoclonal antibody
IOD: integrated optical density
^125^I: Iodine-125
PBS: phosphate buffer solution
PE: phosphatidylethanolamine
PS: phosphatidylserine
RCP: radiochemical purity
T/M: tumor-to-contralateral muscle tissues
TUNEL: terminal deoxynucleotidyl transferase-mediated dUTP nick end labeling
VOI: volume of interest
3PRGD2: 3PEG4-cRGD dimer.
