# Supplementary figures and images for: Treatment of Hepatocellular Carcinoma by Intratumoral Injection of 125I-AA98 mAb and Its Efficacy Assessments by Molecular Imaging
Source: Front Bioeng Biotechnol. 2019 Nov 14;7:319. doi: 10.3389/fbioe.2019.00319 (PMC6868101; doi:10.3389/fbioe.2019.00319)

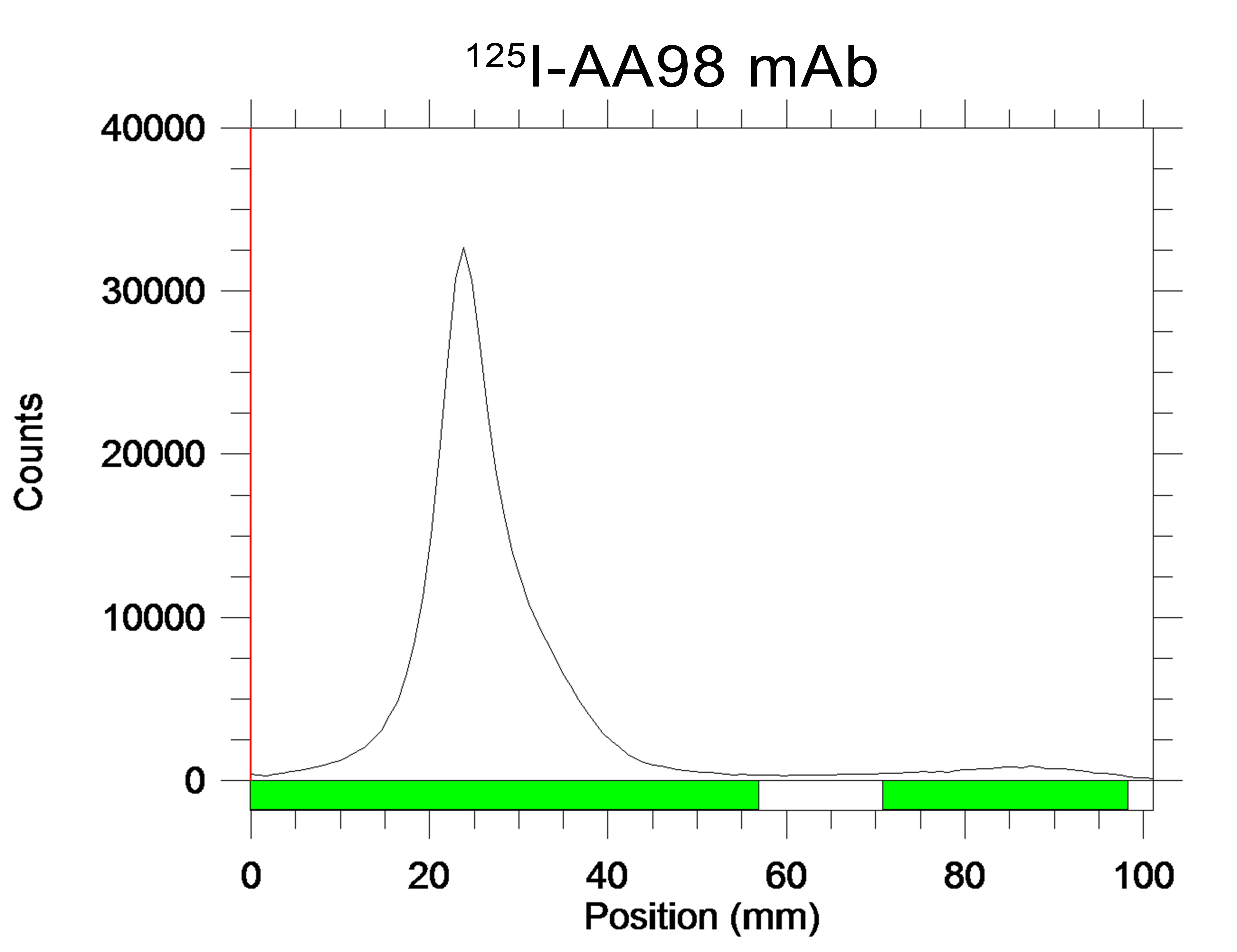

Supplement: Figure S1 — The radiochemical purity (96.3%) of 125I-AA98 mAb was assessed by radio-thin layer chromatography. [file Image_1.TIF]
